# A Coursebook on Aphasia and Other Neurogenic Language Disorders

**DOI:** 10.12669/pjms.41.6.12512

**Published:** 2025-06

**Authors:** Guanqing He, Shaohua Fang, Hui Chang

**Affiliations:** 1 Guanqing He, School of Foreign Languages, Shanghai Jiao Tong University, Shanghai, P. R. China. National Research Centre for Language and Well-being, P.R. China; 2 Shaohua Fang, Department of English, Purdue University, Indiana, USA; 3 Hui Chang, School of Foreign Languages, Shanghai Jiao Tong University, Shanghai, P. R. China. National Research Centre for Language and Well-being, P.R. China

M.N. Hegde. A Coursebook on Aphasia and Other Neurogenic Language Disorders, Fifth Edition. San Diego, CA: Plural Publishing. ISBN: 978-1-63550-422-4.

The fifth edition of *A Coursebook on Aphasia and Other Neurogenic Language Disorders* offers a comprehensive and structured description of aphasia, right hemisphere disorder (RHD), traumatic brain injury (TBI), and dementia—the four major neurogenic language disorders associated with neurological pathologies. As a specialist in fluency disorders, language disorders, research methods, and treatment procedures in communicative disorders, the book author discusses the causes, symptoms, assessment, and treatment of these disorders, reviews theories and literatures from an updated viewpoint, and provides practical recommendations to speech-language pathologists, clinicians, caregivers, and family members. Aphasia is one of the most severe symptoms associated with stroke which is a leading cause of both disability and death worldwide (Saini, 2021). And language and cognition disabilities associated with other neurogenic language disorders are also of significance. Current assessment and treatment of these disabilities, however, fails to exhibit considerable efficacy and beneficial clinical outcomes. A well-rounded book that systematically introduces the disorders with updated knowledge will be essentially helpful for clinical and research professionals devoted to unravelling these problems.

Part-I *Brain and Language* comprises two chapters. The first chapter presents an overview of neuroanatomy and neurophysiology and elucidates brain areas that are involved in various aspects of language production and comprehension at all levels of linguistic representation, from lexicon and syntax to rhythm. It also introduces the brain parts that are involved in cognition functions such as attention and perception. The chapter ends with protective layers of the brain and the cerebral blood supply that are vital to language functions. Chapter-2 surveys the neurodiagnostic methods used to assess language disorders from the oldest post-hoc method to current ones including electroencephalography, magnetoencephalography, and magnetic resonance imaging, among others. This chapter proceeds to delineate the prevalent pathological origins of language disorders with neurological bases and what would happen to the brain structures as a result of these pathologies. Furthermore, this chapter offers a spectrum of guidance for clinicians, family members and caregivers on appropriate responses and interventions in cases of neurologically based language disorders. While the specialized terms and jargons in part I might pose challenges for readers lacking a background in neurology, the discussion is essential for them to understand the content explained in the subsequent parts.

Part-II *Aphasia* departs from the history of scientific study of the disorder and the classic Broca-Wernicke-Lichtheim model. The prevalence, definition, and classification of aphasia are elaborated in Chapter-3. Updated figures are provided to show the current prevalence of aphasia and variations across diverse ethnocultural groups. The author then addresses the contentious topic of aphasic classification. The chapter ends with major symptoms, neuroanatomical bases, and features of the eight aphasia types under classic classification. It also describes cognitive deficits of aphasics including the attention features of transcortical sensory aphasics, among others.

Chapter-4 presents an overview of the assessment process for aphasia, detailing the steps followed since a patient’s admission and outlining the key assessment batteries used to examine different linguistic aspects. This chapter also explores the assessment of functional communication and life quality and elucidates the diagnostic procedures that follow the assessment. Chapter-5 focuses on different aphasia treatments along with their effectiveness and limitations as documented in the literature. It covers the most common behavioral treatment from basic language rehabilitation to social approaches and group treatment that address more complex functional communication skills. High-tech interventions, such as virtual reality therapy and pharmacological options, are briefly introduced as well.

Part-III *Right Hemisphere Disorder* first illustrates the major functions of right hemisphere neuropathology of RHD, followed by a comparison with those of left hemisphere. The symptoms of RHD are then delineated in Chapter 6, encompassing language and affective deficits and cognitive problems which are the most prominent symptoms observed in RHD. The author emphasizes that, different from left hemisphere disorders, quite a few communication deficits due to RHD stem from cognitive issues such as attention, memory, and learning impairments. Chapter 7 starts with the initial screening and assessment tools of RHD, most of which mainly focus on attention and cognitive functions but lack assessment of language skills. Subsequently, the main treatment targets and procedures of RHD are elaborated, each discussed in dedicated sections. These include social communication skills, visual neglect, impaired attention, deficit awareness, and abstract language.

Part-IV *Traumatic Brain Injury* deals with TBL-related issues ranging from the causes and consequences in Chapter 8 to assessment and management in Chapter-9. Chapter 8 stars with the prevalence of TBI across age, gender, and professions, common causes of TBI, and two types of TBI—penetrating and non-penetrating TBI. The chapter elaborates on the immediate neurological impact of TBI at the moment of trauma, followed by the cascade of secondary effects that ensue. It concludes by examining the repercussions of TBI on language, neurobehavioral, cognitive, and executive functions, as well as the subsequent social and vocational challenges. Chapter-9 opens with the initial test and screening soon after hospitalization of TBI patients, followed by an introduction of the main diagnostic assessment procedures and tools. The chapter then outlines the treatment targets and strategies from attention to self-monitoring. Specific training strategies such as alternative and augmentative communication (AAC) devices, skill maintenance program, group therapy, community reentry program, and cognitive rehabilitation are then delineated. The chapter culminates with a discussion on how to sustain treatment outcomes and extend their benefits to daily communication.

Part-V *The Dementias* takes it yet another step forward by introducing the last disorder dementia. Chapter 10 describes the decreasing incidence and increasing prevalence of dementia in varied populations at the outset. Then it divides dementias into reversible and rapidly progressive types and compares their causes and symptoms. Subsequently, the main four types of dementia—dementia of the Alzheimer’s type, vascular dementia, dementia with Lewy bodies, and frontotemporal dementia and subtypes are presented for their incidence, prevalence, risk factors, neuropathology, language disorders, and cognitive deficits in attention, learning, and memory. Chapter-11 concludes with a review of the assessment of dementia. For practical purposes, various management strategies by speech-language pathologists are then nicely examined, including pharmacotherapy and cognitive, behavioral, and communication interventions.

Originally crafted as a coursebook on aphasia and other neurologically based communication disorders at the undergraduate or graduate level, the book has been successful in proving an up-to-date and inconclusive overview of these disorders. The book stands out for its clear structure, going from the neuroanatomy and neurophysiology of the disorders to the symptoms, assessment and treatment procedures for each disorder. With illustrative figures and examples integrated throughout the text, complemented by a comprehensive glossary of cognitive neuroscience terminology at the end of the book, the book achieves both accessibility and engagement for a wide audience.

Another merit of this book is its thorough examination of the cognitive and behavioral features associated with language deficits. It is widely acknowledged that language and cognition are interrelated and cannot be separated (Arendt, 1978). Individuals with various language disorders typically exhibit a cluster of cognitive deficits in the areas of attention, perception, and executive functioning, etc. (Carrion et al., 2018; Jolly et al., 2020). The unique experiences of these patients serve as valuable insights into the workings of the human cognitive system. In this book, the author delves into the brain regions involved in different cognition activities and details the cognitive features of those disorders, such as attention, memory, learning, and reasoning.

While acknowledging the significance of cognitive aspects in these disorders, it is imperative to recognize that current treatments often inadequately address these deficits, particularly in areas where research is lacking. The lack of robust research in this regard will pose challenges for clinicians, particularly in assessing working memory in adults with aphasia. This is partly due to the fragmentation of the literature itself and lack of studies that specifically explored the validity, clinical feasibility, and reliability of working memory tasks (Mayer & Murray, 2012). Addressing these research gaps, the book offers valuable insights for professionals in cognitive psychology and clinical science, urging further exploration of cognitive mechanisms, assessment, and treatment for individuals with language disorders.

The inclusive nature of the volume makes it also a valuable guidance material for clinicians, speech-language pathologists, caregivers, and family members. It offers a comprehensive description of the current practices, including widely used assessment batteries and interventions, and recommendations for how to improve these practices based on critical reviews of theories and research. For instance, the author reiterates the importance of individualized treatment because of differences among individuals diagnosed with the same disorder. The book introduces caregiver programs that equip carers with effective strategies and practical advice for patient care, emphasizing respect and communication by advising against condescending language like elderspeak. This wealth of information also proves beneficial to family members seeking a deeper understanding of the disorder and informed caregiving guidance. Also highlighted in the book is the significance of collaborative efforts involving various stakeholders including both medical and nonmedical specialists, the patients, their family members, and other caregivers. It is evident that the author’s motivation lies in addressing these disorders within a broader context, making the text engaging and relevant to all individuals associated with the disease.

We would highly recommend this book to researchers at all stages of their careers, whether they are junior researchers considering a path in those areas or seasoned professionals seeking a comprehensive understanding of current research limitations and future exploration avenues. Unlike mere cataloging of research findings, the author provides his own insightful reflections, offering a nuanced perspective. Through a meticulous examination of empirical evidence across various topics, the author not only presents a rich body of work but also offers his critical commentaries on the quantity and quality of research. One particularly resonant point made by the author is the caution against overemphasizing the importance of large-scale randomized clinical trials while disregarding or undervaluing single-subject experimental studies.

The book is not without limitations. In the analysis of disorder prevalence across different countries, the current discourse is predominantly centered on data from the United States and other major English-speaking nations This focus on English-centric data extends to the linguistic aspects discussed in the introduction of language features among patients. Incorporating more data from non-English speaking countries would have enriched the discussion and enhanced the generalizability of the research findings because patients with different language backgrounds may exhibit distinct language behaviors albeit for the same feature. Another notable limitation is about the lack of sufficient discussion on working memory. Previous studies have underscored the pivotal role of working memory in both cognitive and language processing. The connections between working memory and language have been substantiated by both behavioral and neurophysiological data across both healthy and language-disordered populations (Mayer & Murray, 2012). This of course remains an area for future inquiries.

While the book has minor issues, they do not overshadow its significant contributions. Overall, it stands as an outstanding coursebook and as a reliable reference for a diverse readership, including clinicians, speech-language pathologists, researchers, caregivers, and family members. For anyone interested in learning about aphasia and neurogenic language disorders from a holistic perspective, this book serves as an ideal starting point.
